# The relationship between diabetes, diabetes-related complications and productive activities among older Europeans

**DOI:** 10.1007/s10198-017-0911-9

**Published:** 2017-06-27

**Authors:** B. Rodriguez-Sanchez, R. J. M. Alessie, T. L. Feenstra, V. Angelini

**Affiliations:** 1 Department of Economics, Econometrics and Finance, Faculty of Economics and Business, Zernike Campus, University of Groningen, Duisenberg Building, Nettelbosje 2, 9747AE Groningen, The Netherlands; 2 Department of Epidemiology, Faculty of Medical Sciences, University Medical Centrum of Groningen, Hanzeplein 1, 9713GZ Groningen, The Netherlands

**Keywords:** Diabetes, Productivity impairment, Fear health limiting work, Volunteering, Complications, Crisis, I00, I10, I15, J01

## Abstract

**Aims:**

To assess the impact of diabetes and diabetes-related complications on two measures of productivity for people in the labour force and out of it, namely “being afraid health limits ability to work before retirement” and “volunteering”.

**Methods and data:**

Logistic regressions were run to test the impact of diabetes and its complications on the probability of being afraid health limits work and being a formal volunteer. The longitudinal sample for the former outcome includes 53,631 observations, clustered in 34,393 individuals, aged 50–65 years old whereas the latter consists of 45,384 observations, grouped in 29,104 individuals aged 65 and above across twelve European countries taken from the Survey of Health, Ageing and Retirement in Europe, from 2006 to 2013.

**Results:**

Diabetes increased the probability of being afraid health limited work by nearly 11% points, adjusted by clinical complications, and reduced the likelihood of being a formal volunteer by 2.7% points, additionally adjusted by mobility problems. We also found that both the probability of being afraid health limits work and the probability of being a formal volunteer increased during and after the crisis. Moreover, having diabetes had a larger effect on being afraid health limits work during the year 2010, possibly related to the financial crisis.

**Conclusions:**

Our findings show that diabetes significantly affects the perception of people regarding the effects of their condition on work, increasing the fear that health limits their ability to work, especially during the crisis year 2010, as well as the participation in volunteering work among retired people.

**Electronic supplementary material:**

The online version of this article (doi:10.1007/s10198-017-0911-9) contains supplementary material, which is available to authorized users.

## Introduction

Studies that analysed the impact of health on productivity [1, 6] concluded that a worse health status increased both measures of productivity impairment, absenteeism and presenteeism, forcing early labour-market exit [7]. In the current analysis, we aim to measure the effect of diabetes on productivity, a disease that mainly affects people in advanced age and, more specifically, one in every four people aged 65 and above [8, 9]. We measure productivity through two different outcomes, depending on the age group: being afraid health limits work for those who are still of working age (up to 65) and being a formal volunteer where people are of retirement age (above 65 years old). In case of the former outcome, only a few studies have looked into the burden of diabetes in terms of productivity impairment [1, 10–16]. For volunteering, less research has been conducted [17–23]: self-perceived health has been found to reduce volunteering rates [24], while only one study showed a negative impact of diabetes on volunteering activities [25].

The lack of studies showing the specific effect of diabetes could be due to the difficulty in measuring the impact of diabetes on productivity, since it is a disease that does not impair an individual’s health directly, but through the complications that it generates. Actually, diabetes can impact productivity in several ways. Firstly, diabetes complications might completely impair the ability to work [10–12], increase the number of days absent from work due to health problems [13–15] or reduce productivity at work [14, 15]. Secondly, individuals with diabetes could be discriminated against at work by their employers due to their concerns about low productivity [16], limiting the type of work they can do. While direct effects on actual productivity are hard to measure, perceptions of patients concerning productivity loss might be another related measure that is affected before actual job loss takes place.

Especially in uncertain economic circumstances, such perceptions may be importantly affected by diseases such as diabetes. We thus also aim to assess the relationship of diabetes and those two productivity measures during the crisis period that recently hit Europe, from 2006 to 2013, which has not been taken into account in any of the studies previously mentioned. During an economic crisis, both insecurity and solidarity might rise. Insecurity could rise due to the instability of employment, whereas the need for helping those who have been heavily affected by the crisis could positively affect solidarity. Observing the trends during that period of time could provide evidence of how relevant health is with respect to productivity in periods of economic uncertainty.

Bearing in mind that the effect of diabetes is generally mediated by its comorbidities, it seems sensible to assess the impact of diabetes on the individual’s perception to perform their work due to health problems and their commitment to volunteering, controlling for diabetes-related complications.

This paper therefore proposes to take a cross-sectional perspective from some European countries to analyse the role of such a prevalent disease, together with some complications, in determining individuals’ commitment to both paid and non-paid work. Having a cross-country data set allows us to control for differences in labour market regulations, which potentially affect the enrolment of people with productive activities, but also country-specific cultural differences.

Our research is of special relevance in order to better determine how programmes and policies should be designed and implemented to ensure and maximize the engagement of old people, in who diabetes is highly prevalent [8, 9], into productive activities.

The following section proposes six hypotheses about the link between diabetes and some diabetes-related and non-related clinical and functional complications with the measures we used to address self-commitment to productive activities. Then, we present the data and methods we use, followed by the analysis of the descriptive and multivariate results. Finally, we discuss the results and conclude.

## Theoretical framework

Some studies have already analysed the association between diabetes and lower productivity, concluding that people with diabetes reported higher numbers of disability days [14, 26–29], lost productivity time [14, 30] and unemployment rates [11–14, 24, 26, 28]. However, they used as outcome measure either the number of working days lost due to diabetes or employment transitions, that is, changing from being employed to be retired or disabled. Not much analysis has evaluated the potential impact of diabetes on work limitations. The study closest to ours is the one by Tunceli et al. [13], who used US data on 7055 employees aged 51–61 years old from the first two waves (1992 and 1994) of the Health and Retirement Study (HRS). Individuals were asked if they had any impairments or health problems at the time of the interview that limited the kind or amount of paid work they could do, which is quite similar to the survey question we are using in this study. Authors concluded that, compared with individuals without diabetes, US men and women with diabetes were 5.4 and 6% points, respectively, more likely to have work limitations. They controlled for health status using two self-reported measures: BMI and the number of other chronic conditions in addition to diabetes (hypertension, heart disease, chronic lung disease, stroke, cancer, arthritis and psychiatric problems). This leads to our first hypothesis:

### Hypothesis 1

Diabetes increases the likelihood of people aged 50–65 years old of being afraid health limits their work, although the magnitude of the effect will be reduced when diabetes-related complications are included in the regression.

Moreover, the aforementioned works tested their hypotheses using data prior to the economic crisis that took place in 2008. In fact, none of them looked at the relevance of time variables. Conversely, we are using data from the time before (2006), during (2010) and after (2013) the crisis period. Moreover, we use a subjective measure (“being afraid that health limits work”) that might be especially sensitive to economic circumstances. Due to the uncertain situation, we expect people to be more afraid of losing their job during and after the crisis than before. One of the factors that could impair an individual’s ability to carry out his/her job appropriately could be health. The economic crisis might force employers to become stricter with regards to the job requirements and employees could experience more pressure while they are in their job position. Hence, people could expect that the fewer limitations they have in their working performance derived from their health status, the less likely it will be that they will lose their job. Thus, our second hypothesis is:

### Hypothesis 2

The fear of health limiting work of people between ages 50 and 65 will increase during 2010 and 2013 with respect to 2006.

As shown in the previous two hypotheses, we expect both diabetes and time variables to be positively associated with being afraid health limits work; we consequently presume the joint effect of both variables will lead to a greater risk of being afraid health limits work. Given the effect of the crisis, which is expected to become apparent in later years, and the impact of diabetes, especially high in old age groups, and impairing individuals’ functioning, we establish our third hypothesis:

### Hypothesis 3

Having diabetes will increase the perception of the individuals aged 50–65 years old being afraid health limits work more during the crisis years 2010 and 2013 with respect to 2006.

Furthermore, non-paid productive activities could be a useful tool for measuring productivity in advanced ages, especially when individuals are retired. According to the existing literature, the likelihood of taking up volunteering seems to be lower at higher ages [19–21], due to their health status, also leading to withdrawals in those who were already performing non-paid work [20]. Nevertheless, not much literature has been found looking specifically at the effect of diabetes [25], leading us to the fourth hypothesis:

### Hypothesis 4

Diabetes will significantly reduce the likelihood of being a formal volunteer (doing charity work), as well as the amount of time dedicated to this task in people aged 65 and older.^1^

Moreover, as a consequence of the economic crisis, rates of volunteering have increased [18–22], showing great differences within European countries [18, 20, 22]. The rationale behind such an increase could be due to a willingness to help people, given the unstable situation, rather than a decision to perform some productive activities as if at work. Moreover, individuals might do charity work because, due to the effect of the crisis, governments could have cut budgets and subsidies for social services, so they may feel that they should do it instead. So, the fifth hypothesis is:

### Hypothesis 5

Volunteering will increase during the years 2010 and 2013 with respect to 2006.

Finally, we aim to analyse the interaction between both independent variables, having diabetes and time, and being a formal volunteer. We expect the interaction term to be significantly related to volunteering, although the sign of its coefficient is uncertain given the opposite interpretations of these variables separately.

### Hypothesis 6

The association between diabetes and volunteering will be different during the crisis period for those older than 65 years old, that is, years 2010 and 2013.

## Methods

### Sample data

Our data are drawn from waves 2, 4 and 5, corresponding to the years 2006/07, 2010 and 2013, respectively, from the Survey of Health, Ageing and Retirement in Europe (SHARE). The survey is a longitudinal survey that aims to provide comprehensive data on socioeconomic characteristics, health and healthcare use, and family networks from multiple European countries and Israel [18].

We limited our analysis to eleven of these countries: Austria, Belgium, Czech Republic, Denmark, France, Germany, Italy, the Netherlands, Spain, Sweden and Switzerland. Other countries were excluded from the analysis because they only appeared in one of the three waves. We then split our sample by age: given that the most common retirement age in Europe is 65 years old, we distinguish between those still of working age (from 50 to 65 years old) and retired individuals^2^ (above 65 years old).

Thus, our population of analysis consisted of 53,631 observations between the ages 50–65 clustered in 34,393 individuals when the outcome was being afraid health limited their work and 45,384 figures grouped in 29,104 individuals when assessing volunteering and aged above 65 years old, that is, those who were already retired.

### Selection of variables

#### Outcome variables

In order to evaluate the fear of health limiting work, we took from SHARE data on the following question: “Are you afraid health limits the kind or amount of work you do?”, to which respondents could choose between “yes” and “no”.

Data on respondents’ commitment to volunteering is based on a question from SHARE that was formulated as follows:

“Have you done any of the following activities in the last month?

1. Done voluntary or charity work
2. Cared for a sick or a disabled adult
3. Provided help to family, friends or neighbours”

According to Hank and Stuck [19], volunteering could be divided into three categories: formal volunteering (having done voluntary or charity work), informal care (care for a sick or disabled adult) and being a carer (provide help to family, friends or neighbours). However, due to changes in question formulation in wave 4, we decided to take the more strict definition of volunteering, formal volunteering.

We then focus on whether respondents have been actively performing volunteering activities, instead of looking at, for example, membership of charity associations. The latter measure, although commonly used, might overestimate the actual engagement, whereas our chosen variable will likely give a more accurate estimation of the real volunteering figures across Europe [22], since respondents are asked about the volunteering done in the last month instead of longer time periods.

We also looked at the frequency of charity work, which could be (1) daily, (2) weekly, and (3) less often than weekly.

#### Independent variables

Since the main independent variable was diabetes, we took self-reported information from SHARE about doctors’ diagnoses on diabetes. Moreover, we also wanted to evaluate the impact of comorbidities on both outcome measures, making a distinction between those related and not related to diabetes. For this, we used information on the following chronic conditions: heart attack, stroke, lung disease, cancer, ulcer, hypertension, and hip fracture. These were the main chronic conditions that were available in SHARE across all waves, as well as all diabetes related conditions that were available in SHARE. We considered the following conditions to be diabetes related: heart attack, stroke, ulcer, and hypertension.

Furthermore, SHARE contains data on the number of mobility problems that individuals might report, ranging from 0, that is, no mobility problems at all, to a maximum of 10. We then generated a dummy variable that took the value 1 if any number of mobility problems had been reported by the respondent and 0 otherwise. This variable was only included when assessing an individual’s engagement in volunteering activities. Mobility problems can be regarded as a health limitation, so its consideration in the analysis of the fear health limited work could lead to estimation problems.

The second main variable of interest was time, so dummy variables for wave 4 (year 2010) and wave 5 (year 2013) were included in the analysis, with wave 2 (year 2006/07) being the reference group.

Moreover, we included sociodemographic factors such as age. For working-age people, we generated dichotomous variables for age groups 50–55, 56–60 and 61–65, the youngest group being the reference group. We did this to control for differences across group ages, since the oldest group might not perceive health as such a big problem as the younger subpopulations, due to their proximity to retirement. On the other hand, we included six age groups, age 65–70, 71–75, 76–80, 81–85, 86–90 and older than 90 years old, in the volunteering analysis. Common to both analyses was the natural logarithm of household income, which was actually our only continuous variable. We also incorporated dummy variables for gender, marital status, and education categories. A detailed explanation can be found in Supplementary Appendix, A1. Finally, we included country dummy variables so we could control for potential differences across countries.

### Statistical analyses

In a first step of analysis, we estimated univariate logit models for the list of covariates and our two binary dependent variables: being afraid health limits work and doing formal volunteering activities.

Then, we estimated a multivariate logistic regression with clustered standard errors, which was actually appropriate when using data from different waves as we did, at the individual level to take into account within individual autocorrelation in the analysis of being afraid health limits work and at the household level when analyzing volunteering, so we took into account correlation between household members’ decisions [31, 32].

Let $\varLambda (t) = e^{t} /(1 + e^{t} )$ be the logistic function with values stretching between zero and one, and let:

1 $$\Pr [{\text{AHLW}}_{ict} = 1|x_{it} ] = \varLambda \left( {\beta_{0} + \beta_{1} SE_{it} + \beta_{2} {\text{diabetes}}_{it} + \beta '_{3} {\text{HI}}_{it} + \gamma_{c} + \zeta_{t} } \right),$$

where *i* represents the individual, *c* country, and *t* year. ${\text{AHLW}}_{ict}$ is a dummy variable indicating that respondent *i* is afraid health limits his/her work in country *c* in year *t*. $x_{it} = ({\text{SE}}_{it} ,{\text{diabetes}}_{it} ,{\text{HI}}_{it} ,{\text{countrydummies}}(\gamma_{c} ),{\text{timedummies}},\zeta_{t} )'$ is a vector of explanatory variables. SE_*it*_, diabetes_*it*_, HI_*it*_ denote the set of socioeconomic variables, having diabetes and chronic conditions, respectively.

Model A adjusted for demographic characteristics, socioeconomic status, chronic conditions not related to diabetes, time and country dummies. In this model some diseases such as cerebrovascular conditions were not included due to their relationship with diabetes. If these complications had been counted in, the gross effect of diabetes would not be measured. However, in order to evaluate the net impact of diabetes on being afraid health limits work, we included clinical complications in Model B. In order to measure the effect of diabetes together with the time dummy on the outcome according to our third and sixth hypotheses, the interaction between these two was included in a last regression (Model C).

The same procedure was followed for our second outcome of interest, to be a formal volunteer, but with an additional inclusion. In Model B, we also looked at the impact of having mobility problems.

After running these three regression models, we tested which model better fits the data using a Wald test. The Wald test compares the null hypothesis that a set of parameters is equal to zero, so, if the test fails to reject the null hypothesis, removing those variables from the model will not substantially damage the fit of such model. We compared Model B against Model A, as the former added clinical complications and mobility problems in case of volunteering to the latter model, and Model C against Model B, testing for the statistical and explanatory relevance of the interactions between diabetes and years 2010 and 2013.

Besides, our aim was also to see how the independent variables affected the different intensities of volunteering. In order to do so, we ran Model C as an ordered logit model. As this kind of model is easier to estimate and interpret than multinomial logit models, it is advantageous to exploit the order nature of the outcomes of the dependent variable [31].

All the statistical analyses were run using Stata 14.

## Results

### Descriptive statistics

Characteristics of the study population by productive outcome are shown in Table 1. Moreover, the table shows a comparison of means test between the two groups.

**Table 1:** Descriptive statistics

Variables	Whole sample (*N* = 53,631)	Afraid health limits work (*N* = 11,259)	Not afraid health limits work (*N* = 42,372)	Univariate analysis	Whole sample (*N* = 45,384)	Formal volunteering (*N* = 8281)	No formal volunteering (*N* = 37,103)	Univariate analysis
Afraid health limits work	20.99%							
Formal volunteering					18.24%			
No formal volunteering					81.76%	–	–	
Less often than weekly					5.61%	30.15%	–	
Weekly					9.05%	51.90%	–	
Daily					3.58%	17.95%	–	
Diabetes	8.81%	16.45%	6.79%	***	15.14%	10.76%	16.12%	***
Age categories				***				***
Age 50–55	25.74%	23.73%	26.27%					
Age 56–60	33.70%	35.03%	33.35%					
Age 61–65	40.56%	41.24%	40.38%					
Age 66–70					31.30%	39.85%	29.40%	
Age 71–75					28.16%	31.78%	27.36%	
Age 76–80					20.66%	18.13%	21.22%	
Age 81 to 85					12.67%	7.46%	13.83%	
Age 86–90					5.62%	2.38%	6.35%	
Age older than 90					1.57%	0.37%	1.83%	
Female	56.00%	56.66%	55.82%		54.25%	51.25%	54.92%	
Education categories				***				***
Low education	31.6%	38.81%	29.25%		45.15%	32.18%	48.04%	
Medium education	40.28%	39.00%	40.62%		30.11%	35.55%	28.90%	
High education	25.96%	18.28%	28.00%		18.09%	30.67%	15.28%	
Marital status categories				***				***
Non-single	75.11%	69.20%	76.68%		64.95%	68.69%	64.11%	
Never married	6.90%	8.22%	6.54%		4.01%	4.53%	3.90%	
Separated, divorced or widowed	17.12%	21.59%	15.93%		29.01%	25.26%	29.85%	
Mean (SD) Log-household income	10.33 (1.11)	10.09 (1.09)	10.39 (1.11)	***	10.12 (1.05)	10.42 (0.91)	10.05 (1.07)	***
Chronic lung disease	5.04%	11.72%	3.27%	***	7.36%	5.59%	7.75%	***
Cancer	3.92%	8.86%	2.61%	***	6.48%	6.63%	6.45%	
Ulcer	3.51%	7.19%	2.54%	***	4.12%	3.67%	4.23%	***
Heart attack	5.90%	14.15%	3.71%	***	15.96%	13.37%	16.54%	***
Hypertension	29.96%	38.61%	27.66%	***	45.70%	40.45%	46.87%	***
Stroke	2.09%	6.32%	0.97%	***	4.99%	3.21%	5.38%	***
Hip fracture	0.96%	2.42%	0.58%	***	2.89%	2.11%	3.07%	***
Mobility problems					59.57%	47.06%	62.36%	***
Countries				***				***
Austria	8.74%	6.79%	9.26%		9.36%	9.12%	9.42%	
Germany	8.27%	11.16%	7.51%		7.07%	8.21%	6.81%	
Sweden	5.91%	5.90%	5.92%		9.16%	9.01%	9.19%	
The Netherlands	8.23%	9.06%	8.01%		7.38%	14.79%	5.72%	
Spain	9.20%	8.66%	9.35%		12.32%	2.54%	14.51%	
Italy	8.72%	5.38%	9.61%		9.83%	5.77%	10.73%	
France	10.92%	11.19%	10.85%		9.94%	11.65%	9.56%	
Denmark	8.19%	9.83%	7.76%		6.69%	10.34%	5.88%	
Switzerland	7.22%	4.09%	8.05%		6.78%	10.84%	5.87%	
Belgium	12.87%	13.84%	12.61%		10.54%	13.42%	9.90%	
Czech Republic	11.73%	14.10%	11.09%		10.93%	4.31%	12.41%	
Waves								
Wave 2 (years 2006/07)	16.39%	16.57%	16.35%		13.58%	12.23%	13.88%	
Wave 4 (year 2010)	36.78%	36.33%	36.89%		32.79%	36.00%	32.08%	
Wave 5 (year 2013)	46.83%	47.09%	46.76%		53.63%	51.77%	54.04%	

Percentages are presented referring to the mean percentage between waves, unless indicated otherwise

Comparison of means tests cluster observations at the individual level in case of being afraid health limits work and at the household level in case of formal volunteering

*** *p* < 0.01, ** *p* < 0.05

With regards to those being afraid health limits their work, diabetes prevalence more than doubles between groups (16.45% in people being afraid vs 6.79% in case of not being afraid of health limiting work). The same pattern holds for the other chronic conditions. For example, 11.72, 14.15, 38.61 and 6.32% of those being afraid health limits work suffer from chronic lung disease, heart attack, hypertension or stroke, respectively, in contrast with their comparison group, for whom these rates drop to 3.27, 3.71, 27.66 and 0.97%, respectively. In addition, differences between waves are not significant. Those who are afraid health limits their work are slightly older, with a lower education level, with no current partner, and with lower income.

In terms of volunteering, significant differences have also been found between those doing charity work and those not. Those not being formal volunteers had higher rates of diabetes prevalence than their counterparts (16.12 vs 10.76%). The same pattern holds for the other chronic conditions and mobility problems. For example, 16.54, 46.87 and 62.36% of the non-volunteers have heart attack, hypertension or mobility problems, respectively, in comparison to the volunteers, whose ratios decrease to 13.37, 40.45 and 47.06%, respectively. In addition, the three time variables report no significant relationship with volunteering. Those who do not provide charity work are older, more likely to be women, with lower education, with no current partner, and with lower income.

Regarding both outcomes, country dummies also capture differences in reporting styles, which presumably differ across countries. With respect to diabetes prevalence, some differences can also be observed between countries (Table 2). Diabetes prevalence is highest in the Czech Republic and Spain, with percentages of 20–23% of the population older than 65 years old. Lowest diabetes prevalence can be observed in Denmark and Switzerland, which barely get to 10% for people above 65 years old.

**Table 2:** Country specific data, by outcome

Countries	Whole sample		Afraid health limits work		Univariate analysis	Formal volunteering		Univariate analysis
	*N*	Diabetes prevalence *N* (%)	*N*	Diabetes prevalence *N* (%)		*N*	Diabetes prevalence *N* (%)	
Austria	8934	1030 (11.53%)	4686	447 (9.54%)	***	4248	583 (13.72%)	**
Germany	7645	1005 (13.15%)	4437	434 (9.78%)	***	3208	571 (17.80%)	***
Sweden	7327	766 (10.45%)	3171	253 (7.98%)	***	4156	513 (12.34%)	
The Netherlands	7763	765 (9.85%)	4414	359 (8.13%)	***	3349	406 (12.12%)	***
Spain	10,530	1674 (15.90%)	4936	511 (10.35%)	**	5594	1163 (20.79%)	***
Italy	9136	1089 (11.92%)	4676	391 (8.36%)	***	4460	698 (15.65%)	***
France	10,366	1144 (11.04%)	5856	538 (9.19%)	***	4510	606 (13.44%)	***
Denmark	7433	557 (7.49%)	4395	229 (5.21%)	***	3038	328 (10.80%)	***
Switzerland	6946	459 (6.61%)	3870	196 (5.06%)	***	3076	263 (8.55%)	***
Belgium	11,684	1197 (10.24%)	6901	594 (8.61%)	***	4783	603 (12.61%)	***
Czech Republic	11,251	1912 (16.99%)	6289	775 (12.32%)	***	4962	1137 (22.91%)	***
Overall sample	99,015	11,575 (11.69%)	53,631	4344 (8.81%)	–	45,384	6871 (15.14%)	–

Comparison of means tests cluster observations at the individual level in case of being afraid health limits work and at the household level in case of formal volunteering

*** *p* < 0.01, ** *p* < 0.05

### Results from the multivariate regressions

#### Being afraid health limits work

Tables 3 and 4 report the results for the overall sample for the outcome “being afraid health limits work”. Having diabetes significantly increases the risk of being afraid health limits work, although its coefficient drops from 0.902 in Model A to 0.704 in Model B, when the regression is adjusted for clinical complications (Table 3). Stroke and hip fracture are the main complications increasing the likelihood of reporting being afraid health limits work.

**Table 3:** Results from the logit regressions regarding the fear of health limiting work for the overall sample

Variables	Average marginal effects Model A	Average marginal effects Model B	Average marginal effects Model C
Diabetes	0.162***	0.116***	0.101***
	(0.008)	(0.008)	(0.006)
Diabetes# Wave4			0.127**
			(0.011)
Diabetes# Wave5			0.111
			(0.010)
Chronic lung disease	0.232***	0.195***	0.156***
	(0.011)	(0.011)	(0.007)
Cancer	0.252***	0.235***	0.183***
	(0.012)	(0.012)	(0.008)
Ulcer		0.141***	0.118***
		(0.012)	(0.009)
Heart attack		0.212***	0.167***
		(0.011)	(0.007)
Hypertension		0.037***	0.036***
		(0.004)	(0.004)
Stroke		0.316***	0.234***
		(0.018)	(0.012)
Hip fracture		0.257***	0.197***
		(0.024)	(0.016)
Austria	−0.100***	−0.099***	−0.117***
	(0.007)	(0.007)	(0.010)
Sweden	−0.043***	−0.038***	−0.040***
	(0.010)	(0.009)	(0.011)
The Netherlands	−0.045***	−0.037***	−0.039***
	(0.009)	(0.009)	(0.010)
Spain	−0.096***	−0.084***	−0.097***
	(0.007)	(0.007)	(0.009)
Italy	−0.138***	−0.130***	−0.168***
	(0.006)	(0.006)	(0.010)
France	−0.058***	−0.052***	−0.056***
	(0.008)	(0.008)	(0.009)
Denmark	0.010	0.014	0.013
	(0.010)	(0.010)	(0.009)
Switzerland	−0.113***	−0.101***	−0.123***
	(0.007)	(0.007)	(0.011)
Belgium	−0.045***	−0.045***	−0.047***
	(0.008)	(0.007)	(0.009)
Czech Republic	−0.076***	−0.077***	−0.086***
	(0.007)	(0.007)	(0.009)
Wave 4 (2010)	0.011***	0.011***	0.011***
	(0.004)	(0.004)	(0.004)
Wave 5 (2013)	0.010**	0.013***	0.013***
	(0.005)	(0.005)	(0.005)
N (observations)	53,631	53,631	53,631
N (clusters)	34,393	34,393	34,393
Log pseudolikelihood	−25,349.10	−24,289.65	−24,287.48
Wald chi^2^	2733.22	3627.48	3634.48
Prob > chi^2^	0.000	0.000	0.000

Clustered standard errors at individual level in parentheses

Reference categories: age 50–55, medium education, being separated/divorced/widowed, Germany and wave 2 (year 2006/07)

In every specification, we control for age, gender, education, marital status and household income

Model A includes diabetes, sociodemographic characteristics (age, gender, education, marital status and household income), and non-diabetes related complications (chronic lung disease and cancer). Model B adds to the previous model diabetes-related clinical complications: ulcer, heart attack, hypertension, stroke and hip fracture. Model C includes the above variables and the interactions between the main disease of interest, diabetes, and the time variables, wave 4 (year 2010) and wave 5 (year 2013)

*** *p* < 0.01, ** *p* < 0.05

**Table 4:** Average marginal effects of clinical and functional complications if individuals have diabetes from the logistic regressions

Variables	Average marginal effects Model A	Average marginal effects Model B	Average marginal effects Model C
No chronic lung disease	0.161***	0.115***	0.099***
	(0.008)	(0.008)	(0.006)
Chronic lung disease	0.208***	0.156***	0.149***
	(0.009)	(0.010)	(0.009)
No cancer	0.161***	0.115***	0.099***
	(0.008)	(0.008)	(0.006)
Cancer	0.206***	0.157***	0.153***
	(0.009)	(0.010)	(0.009)
No ulcer		0.116***	0.100***
		(0.008)	(0.006)
Ulcer		0.150***	0.139***
		(0.010)	(0.009)
No heart attack		0.115***	0.099***
		(0.008)	(0.006)
Heart attack		0.157***	0.151***
		(0.010)	(0.009)
No hypertension		0.113***	0.097***
		(0.008)	(0.006)
Hypertension		0.125***	0.110***
		(0.008)	(0.007)
No stroke		0.116***	0.100***
		(0.008)	(0.006)
Stroke		0.154***	0.157***
		(0.009)	(0.009)
No hip fracture		0.116***	0.100***
		(0.008)	(0.006)
Hip fracture		0.156***	0.154***
		(0.009)	(0.009)
*N* (observations)	53,631	53,631	53,631

Clustered standard errors at individual level in parentheses

The coefficient on not having each disease denotes the individual effect of having diabetes on the outcome. The coefficient on having each disease represents the joint effect of having diabetes and each condition on the probability of being afraid health limits work

*** *p* < 0.01, ** *p* < 0.05

Looking at the marginal effect of diabetes, we see that having diabetes increases the probability of reporting being afraid by 0.116 in Model B, slightly lower than in Model A, when its marginal effect without diabetes-related complications included is 0.162. Moreover, the relevance of diabetes and its comorbidities is shown in Table 4, which shows the average marginal effect of each specific complication together with diabetes compared to not suffering from any of them. The greatest burden of the listed complications, jointly with diabetes, is given by cancer and heart attack (0.157 in both cases).

In addition, regarding the time variables, waves 4 and 5 emerge as significant variables in both regression Models, A and B. Being in the year 2010 increases the probability of reporting being afraid by 0.011 in both regression models, whereas living in the year 2013 increases such probability by 0.010 in Model A, but 0.013% points in Model B, with respect to wave 2, that is, year 2006/07. Besides, the interaction between diabetes and wave 4 is significant and positively associated with the outcome, increasing the probability of the fear by 0.643 (Model C, Table 3). Contrariwise, the interaction between diabetes and wave 5 is not significant.^3^

Some differences can also be observed across countries. Denmark is the only country reporting a positive association with the outcome, meaning that the Danish subsample is more afraid health limits the amount or type of work they can do then the German population, which is the reference category (average marginal effect, 0.013 in Model C). On the other hand, living in Italy reduces the risk of being afraid health limits work the most (average marginal effect, −0.168), compared to Germany.

#### Formal volunteering: charity work

Tables 5, 6 and Supplementary Table A2, Appendix report the results for the outcome “formal volunteering: charity work”. Having diabetes significantly reduces the probability of being a formal volunteer. Its marginal effect is −0.261 in Model A and −0.209 in Model B, when the regression is adjusted for clinical complications and mobility problems (Table 5). Actually, stroke and mobility problems are the main complications that significantly reduce the most the probability of doing charity work. Moreover, if we look at the marginal effect of diabetes in the outcome (Table 5), we see that people with diabetes are 0.027% points less likely to be formal volunteers in Model B. Moreover, the relevance of diabetes and its comorbidities is shown in Table 6. Comorbidities and mobility problems reduce its marginal effect when, in addition, respondents have diabetes.

**Table 5:** Results from the logit regressions regarding formal volunteering for the overall sample

VARIABLES	Average marginal effects Model A	Average marginal effects Model B	Average marginal effects Model C
Diabetes	−0.033***	−0.027***	−0.028***
	(0.006)	(0.006)	(0.007)
Diabetes# Wave4			−0.002
			(0.008)
Diabetes# Wave5			−0.040
			(0.006)
Chronic lung disease	−0.037***	−0.030***	−0.032***
	(0.007)	(0.007)	(0.008)
Ulcer		0.010	0.010
		(0.010)	(0.010)
Heart attack		−0.001	−0.001
		(0.006)	(0.006)
Hypertension		−0.005	−0.005
		(0.004)	(0.004)
Stroke		−0.036***	−0.039***
		(0.009)	(0.010)
Hip fracture		−0.005	−0.004
		(0.012)	(0.012)
Mobility problems		−0.034***	−0.034***
		(0.005)	(0.005)
Austria	−0.029***	−0.029***	−0.031***
	(0.010)	(0.010)	(0.011)
Sweden	−0.024**	−0.027***	−0.028**
	(0.010)	(0.010)	(0.011)
The Netherlands	0.148***	0.141***	0.118***
	(0.014)	(0.014)	(0.010)
Spain	−0.144***	−0.145***	−0.212***
	(0.006)	(0.006)	(0.015)
Italy	−0.061***	−0.061***	−0.068***
	(0.010)	(0.010)	(0.012)
France	0.027**	0.026**	0.025**
	(0.011)	(0.011)	(0.011)
Denmark	0.051***	0.046***	0.043***
	(0.013)	(0.013)	(0.011)
Switzerland	0.058***	0.052***	0.048***
	(0.013)	(0.013)	(0.011)
Belgium	0.032***	0.032***	0.031***
	(0.011)	(0.011)	(0.010)
Czech Republic	−0.113***	−0.111***	−0.141***
	(0.007)	(0.007)	(0.012)
Wave 4 (2010)	0.060***	0.056***	0.054***
	(0.006)	(0.006)	(0.005)
Wave 5 (2013)	0.048***	0.038***	0.038***
	(0.007)	(0.007)	(0.007)
*N* (observations)	45,384	45,384	45,384
*N* (clusters)	18,647	18,647	18,647
Log pseudolikelihood	−19,224.71	−19,035.37	−19,035.27
Wald chi^2^	2262.79	2437.11	2439.53
Prob > chi^2^	0.000	0.000	0.000

Clustered standard errors at household level in parentheses. Reference categories: age 65–70, medium education, being separated/divorced/widowed, Germany and wave 2 (year 2006/07)

In every specification, we control for age, gender, education, marital status and household income

Model A includes diabetes, sociodemographic characteristics (age, gender, education, marital status and household income), and non-diabetes related complications (chronic lung disease). Model B adds to the previous model diabetes-related clinical complications: ulcer, heart attack, hypertension, stroke, hip fracture and mobility problems. Model C includes the above variables and the interactions between the main disease of interest, diabetes, and the time variables, wave 4 (year 2010) and wave 5 (year 2013)

*** *p* < 0.01, ** *p* < 0.05

**Table 6:** Average marginal effects of clinical and functional complications if individuals have diabetes from the logistic regressions

Variables	Average marginal effects Model A	Average marginal effects Model B	Average marginal effects Model C
No chronic lung disease	−0.033***	−0.027***	−0.028***
	(0.008)	(0.006)	(0.007)
Chronic lung disease	−0.029***	−0.024***	−0.025***
	(0.007)	(0.005)	(0.006)
No ulcer		−0.027***	−0.028***
		(0.006)	(0.007)
Ulcer		−0.028***	−0.029***
		(0.006)	(0.007)
No heart attack		−0.027***	−0.028***
		(0.006)	(0.007)
Heart attack		−0.027***	−0.028***
		(0.006)	(0.007)
No hypertension		−0.027***	−0.028***
		(0.006)	(0.007)
Hypertension		−0.027***	−0.028***
		(0.006)	(0.007)
No stroke		−0.027***	−0.028***
		(0.006)	(0.007)
Stroke		−0.023***	−0.024***
		(0.005)	(0.006)
No hip fracture		−0.027***	−0.028***
		(0.006)	(0.007)
Hip fracture		−0.026***	−0.027***
		(0.006)	(0.007)
No mobility problems		−0.029***	−0.030***
		(0.007)	(0.007)
Mobility problems		−0.025***	−0.026***
		(0.006)	(0.006)
*N* (observations)	45,384	45,384	45,384

Clustered standard errors at household level in parentheses

The coefficient on not having each disease denotes the individual effect of having diabetes on the outcome. The coefficient on having each disease represents the joint effect of having diabetes and each condition on the probability of being a formal volunteer

*** *p* < 0.01, ** *p* < 0.05

In addition, both time variables are always significant across regression models, reducing its coefficient when all clinical and mobility problems are included (coefficient 0.431 in Model A and 0.406 in Model B in the case of wave 4 and 0.362 in Model A and 0.287 in Model B in the case of wave 5). Actually, their average marginal effects on the outcome are 0.056 and 0.038 for waves 4 and 5, respectively, in Model B, increasing the likelihood of doing charity work. Furthermore, the interactions between diabetes and time are not significant.^4^

Some differences can also be observed across countries. Southern countries, such as Italy and Spain report negative coefficients, signaling that living in any of these countries reduces the probability of being a formal volunteer, in comparison to Germany, the reference category, as well as in Austria, Sweden or the Czech Republic. The rest of the countries included in the analysis report positive coefficients.

Supplementary Table A2 in the Appendix also shows that diabetes significantly reduces the amount of formal volunteering provided. The probability of not doing any amount of formal volunteering is increased by 0.023 if the individual has diabetes and this disease reduces the likelihood of doing charity work daily, weekly or less often than that by 0.006, 0.013 and 0.006, respectively.

## Discussion

In this analysis we aimed to assess the relationship between diabetes and productivity using European data for three different periods around the financial crisis. We used two measures of productivity, depending on age: for those of working age, that is, from 50 to 65 years old, we employed being afraid health limited their work as the outcome; and for the individuals above 65, we modelled productivity through volunteering activities.

This study showed that diabetes increases the likelihood people aged 50–65 years old reported being afraid health limits work by 16% points, falling to 12, after controlling for clinical complications, suggesting a positive relationship between diabetes and the fear of health limiting work in people still of working age. In addition, our results suggest that the fear of health limiting work increased during the years after the crisis, 2010 and 2013, with respect to the time before the crisis, 2006, even after including clinical complications. This could reflect the increased uncertainty of the employment situation after the economic crisis. In our third hypothesis, we expected that the fear of health limiting work of people of working age with diabetes was higher in the years 2010 and 2013, compared to 2006. Our hypothesis was only confirmed in the case of the interaction between diabetes and year 2010, increasing the probability of being afraid by 13 percentage points, but no significant results were found for 2013. This result might be driven by the combination of the impairing effect of diabetes together with the fact that the economic crisis hit stronger in the early years of the crisis, leading to a greater fear of limiting the individual’s performance at work.

Regarding volunteering in people older than 65 years old, it was shown that having diabetes reduces the likelihood of performing volunteering work by about 3% points in comparison to those people without diabetes, as well as reducing the frequency of carrying out such activity. Year 2010 increased the probability of doing charity work by 0.06, which is larger than the average marginal effect of the year 2013, 0.04, even after adjusting by clinical complications and mobility problems. The rationale behind such increase might be greater solidarity or greater need for charity work rather than the individual willingness to be productive. Finally, our results do not support our last hypothesis about the joint effect of having diabetes in people aged 65 and above in the year 2010 and 2013 on doing charity work.

Moreover, some differences have been observed across countries. With regards to our first outcome, only Denmark reported to increase significantly the likelihood of being afraid health limited the amount or type of work one can do in comparison to Germany, whereas a negative association between Italy, Spain, Austria, Sweden and the Czech Republic and volunteering was displayed, also compared to the German population. However, these differences should be interpreted with caution in case of both outcomes given the potential differences existing in their reporting styles [33]. With respect to being afraid health limits work, a reporting bias could be present due to the culture and the specific characteristics of each country; and also regarding the second outcome, since it has been shown that, especially in Southern countries [34], the frequency of volunteering has increased more than in other areas, but maybe not the amount of people doing so.

Therefore, our results about the first outcome support those obtained by Tunceli et al. [13], though we use a different outcome measure. The main driver behind the difference in size of the effects can be the subjective feature of the outcome used in the present paper, being afraid health limits work, compared to the objective character of the question used by Tunceli et al., whether the individuals had any impairments or health problems that limited work. Hence, latter respondents report actual health problems that impair work, whereas former individuals report their personal perceptions. Moreover, this variation can also stem from the increase in diabetes prevalence or from the difference in the composition of the sample and the time selection. Tunceli et al. [13] used US data from 7055 respondents from 1992 to 1994 and our results are driven by 34,393 individuals from 2006 to 2013. Another explanation could be the different reporting style between the United States and some countries, such as those in Europe, as the literature has already shown [35]. Moreover, our results regarding volunteering activities and diabetes confirm the findings of the American Diabetes Association (2008) [25], who included volunteering within the productivity measure of those not in the labour force. However, the single analysis between diabetes and volunteering is not available in the published document, so we cannot compare the magnitude of our results to theirs.

Some limitations should also be mentioned. First of all, our measure of productivity for those in the workforce did not include the number of days lost due to health or reduced productivity at work, which are the most common measures of productivity losses. This kind of information was not available in the data set used, so we took being afraid health limits work as one of the main outcomes in the study, which, as it is subjective, can be very sensitive to changes in an individual’s situation. Individual perception about his/her ability to perform some activities due to health problems has previously been used in the literature [36]. Authors aimed to assess the relationship between health and retirement in the United Kingdom. They built their health main measure from two different health-related measures: having certain health problems and difficulties, and feeling that their health limits their ability to perform certain daily activities. The latter is a close measure to the subjective outcome we use in the analysis, which supports the use of individual’s feelings together with other more objective health measures. However, the interpretation of the results in the current analysis could lead to lower or higher productivity. For example, it is clear that if individual health gets worse, being diagnosed as having diabetes in this case, the fear of health limiting work is going to increase and also productivity decreases due to health problems. However, when interpreting the association between the time variables and the outcome, an increase of the fear could also result in higher productivity so as to prove that the individual should not be fired. Second, we could not obtain information on other types of volunteering, care for family or informal care, which were available in wave 2, but some changes were made to the question in wave 4. So, we could only stick to the strictest definition of volunteering, which refers to charity work [18]. Thirdly, due to data restrictions, we excluded seven countries from the analysis from the nineteen countries that SHARE provides information from. However, the results reported in this study are still accurate since we included a representative sample of the European population. Finally, the self-reported feature of the data, especially health conditions, could bias the results, since it could lead to recall bias and, hence, the results here could over- or underestimate the true impact of diabetes. Nevertheless, there are several findings showing the reliability of data from health conditions collected using self-reported information [37, 38].

Our results contribute to the literature in three ways. First, much has been written regarding the association between diabetes and number of days lost due to health reasons or reduced productivity at work, but little is known about the relationship with being afraid health limits work and non-paid productivity measures, such as volunteering. Second, not all the existing studies have included diabetes-related clinical and functional complications. Actually, one study showed that, by excluding those, we could underestimate the impact of diabetes [10]. Finally, we have also assessed the influence of uncertain economic periods, which has not been done before, and its association with both productivity alone and jointly with diabetes. While three waves is a relatively modest number, the observed patterns over the years 2006, 2010 and 2013 are suggestive for the effect of an uncertain economic situation on both subjective (fear of health limiting work) and objective (volunteering participation) measures of the impact of diabetes on productivity. Our results provide evidence that diabetes affects patients, employers, and society by contributing to work loss through work limitations and decreases in volunteering activities, even in unstable environments, such as the economic crisis that hit Europe recently. Moreover, the economic burden associated with diabetes is likely to increase as diabetes becomes more prevalent.

## Conclusion

This study has shown that, within those aged between 50 and 65 years, diabetes significantly impacts individuals’ perception about their ability to work, increasing the fear that health limits their ability to work, although its burden is mediated by diabetes-related clinical complications. Moreover, the impact of diabetes increased after the economic crisis hit, as shown in the year 2010. Furthermore, diabetes also hampers participation in volunteering activities for those of retirement age, reducing the probability of doing charity work. However, no significant effects have been found regarding the joint effect of suffering from diabetes and the years after the economic crisis on volunteering activities.

## Electronic supplementary material

Below is the link to the electronic supplementary material.

Supplementary material 1 (DOCX 22 kb)

## Footnotes

1 We did not include individuals younger than 65 in the analysis of volunteering since it could be considered as a substitute for a paid job, which would complicate the analysis.

2 According to the responses to the employment status question from the survey, only 2% of the people aged 65+ are employed or self-employed, whereas almost 85% are retired and another 11.5% declared themselves as homemakers.

3 Moreover, the Wald test confirms that the diabetes and time dummies are jointly significant and Model C is the model that better fits the data.

4 Moreover, the Wald test confirms that the interactions are not jointly significant, so that Model B is thus the best for explaining the association between the set of explanatory variables and the outcome.
