# Supplementary material for: The relationship between diabetes, diabetes-related complications and productive activities among older Europeans
Source: Eur J Health Econ. 2017 Jun 27;19(5):719–34. doi: 10.1007/s10198-017-0911-9 (PMC5948290; doi:10.1007/s10198-017-0911-9)
Supplement: Supplementary file 1 — Supplementary material 1 (DOCX 22 kb) [file 10198_2017_911_MOESM1_ESM.docx]

ARTICLE TITLE: THE RELATIONSHIP BETWEEN DIABETES, DIABETES-RELATED COMPLICATIONS AND PRODUCTIVE ACTIVITIES AMONG OLDER EUROPEANS

JOURNAL TITLE: THE EUROPEAN JOURNAL OF HEALTH ECONOMICS

APPENDIX

A1: List of variables included in the analysis

| **Variable** | **Coding** |
| --- | --- |
| Being afraid health limits work | 1: respondent has been afraid health limited the kind or amount of work s/he did; 0: otherwise |
| Formal volunteering | 1: respondent has done voluntary or charity work in the last month; 0: otherwise |
| Age 50 - 55 | 1: respondent’s age lies between 50 and 55 years old; 0: otherwise |
| Age 56 – 60 | 1: respondent’s age lies between 56 and 60 years old; 0: otherwise |
| Age 61 – 65 | 1: respondent’s age lies between 61 and 65 years old; 0: otherwise |
| Age 66 – 70 | 1: respondent’s age lies between 66 and 70 years old; 0: otherwise |
| Age 71 - 75 | 1: respondent’s age lies between 71 and 75 years old; 0: otherwise |
| Age 76 – 80 | 1: respondent’s age lies between 76 and 80 years old; 0: otherwise |
| Age 81 – 85 | 1: respondent’s age lies between 81 and 85 years old; 0: otherwise |
| Age 86 - 90 | 1: respondent’s age lies between 86 and 90 years old; 0: otherwise |
| Age 90+ | 1: respondent is older than 90; 0: otherwise |
| Female | 1: female; 0: male |
| Low education | 1: respondent had completed primary education or first stage of basic education and lower secondary or second stage of basic education; 0: otherwise |
| Medium education | 1: respondent had completed (upper) secondary education and post-secondary non-tertiary education; 0: otherwise |
| High education | 1: respondent had completed first and second stage of tertiary education; 0: otherwise |
| Non-single | 1: married or with a registered partner; 0: otheriwse |
| Never married | 1: never married; 0: otherwise |
| Separated, divorced or widowed | 1: separated, divorced or widowed; 0: otherwise |
| Log household income | 1: respondent’s income is in the lowest quintile group; 0: otherwise |
| Chronic lung disease | 1: respondent has any chronic lung disease; 0: otherwise |
| Cancer | 1: respondent has cancer; 0: otherwise |
| Ulcer | 1: respondent has an ulcer; 0: otherwise |
| Heart attack | 1: respondent has had a heart attack; 0: otherwise |
| Hypertension | 1: respondent has hypertension; 0: otherwise |
| Stroke | 1: respondent has had a stroke; 0: otherwise |
| Hip fracture | 1: respondent has had a hip fracture; 0: otherwise |
| Mobility problems | 1: any number of mobility problems had been reported by the respondent; 0: otherwise |
| Austria | 1: respondent lives in Austria; 0: otherwise |
| Germany | 1: respondent lives in Germany; 0: otherwise |
| Sweden | 1: respondent lives in Sweden; 0: otherwise |
| The Netherlands | 1: respondent lives in The Netherlands; 0: otherwise |
| Spain | 1: respondent lives in Spain; 0: otherwise |
| Italy | 1: respondent lives in Italy; 0: otherwise |
| France | 1: respondent lives in France; 0: otherwise |
| Denmark | 1: respondent lives in Denmark; 0: otherwise |
| Greece | 1: respondent lives in Greece; 0: otherwise |
| Switzerland | 1: respondent lives in Switzerland; 0: otherwise |
| Belgium | 1: respondent lives in Belgium; 0: otherwise |
| Czech Republic | 1: respondent lives in Czech Republic; 0: otherwise |
| Wave 2 (years 2006/07) | 1: data was collected from wave 1; 0: otherwise |
| Wave 4 (year 2010) | 1: data was collected from wave 2; 0: otherwise |
| Wave 5(year 2013) | 1: data was collected from wave 4; 0: otherwise |

A2: Average marginal effects from the ordered logit regression regarding frequency of formal volunteering for the overall sample

| VARIABLES | Average marginal effects: no formal volunteering | Average marginal effects: less often than weekly | Average marginal effects: weekly | Average marginal effects: daily |
| --- | --- | --- | --- | --- |
| Diabetes | 0.0250*** | -0.00602*** | -0.0133*** | -0.00568*** |
|  | (0.00645) | (0.00155) | (0.00344) | (0.00147) |
| Chronic lung disease | 0.0276*** | -0.00664*** | -0.0147*** | -0.00624*** |
|  | (0.00797) | (0.00193) | (0.00425) | (0.00182) |
| Ulcer | -0.00757 | 0.00182 | 0.00403 | 0.00171 |
|  | (0.00981) | (0.00236) | (0.00523) | (0.00222) |
| Heart attack | 0.000967 | -0.000233 | -0.000515 | -0.000219 |
|  | (0.00533) | (0.00128) | (0.00284) | (0.00121) |
| Hypertension | 0.00723 | -0.00174 | -0.00385 | -0.00164 |
|  | (0.00398) | (0.000959) | (0.00212) | (0.000904) |
| Stroke | 0.0358*** | -0.00862*** | -0.0191*** | -0.00811*** |
|  | (0.00974) | (0.00235) | (0.00520) | (0.00222) |
| Hip fracture | 0.00756 | -0.00182 | -0.00403 | -0.00171 |
|  | (0.0117) | (0.00282) | (0.00624) | (0.00265) |
| Mobility problems | 0.0327*** | -0.00788*** | -0.0174*** | -0.00741*** |
|  | (0.00452) | (0.00110) | (0.00242) | (0.00105) |
| Austria | 0.0454*** | -0.0109*** | -0.0242*** | -0.0103*** |
|  | (0.0101) | (0.00242) | (0.00536) | (0.00231) |
| Sweden | 0.0289*** | -0.00695*** | -0.0154*** | -0.00654*** |
|  | (0.0103) | (0.00248) | (0.00549) | (0.00234) |
| The Netherlands | -0.110*** | 0.0264*** | 0.0584*** | 0.0248*** |
|  | (0.00957) | (0.00234) | (0.00519) | (0.00229) |
| Spain | 0.211*** | -0.0507*** | -0.112*** | -0.0477*** |
|  | (0.0149) | (0.00373) | (0.00816) | (0.00362) |
| Italy | 0.0622*** | -0.0150*** | -0.0332*** | -0.0141*** |
|  | (0.0119) | (0.00288) | (0.00635) | (0.00269) |
| France | -0.0188 | 0.00452 | 0.01000 | 0.00425 |
|  | (0.0100) | (0.00242) | (0.00535) | (0.00228) |
| Denmark | -0.0364*** | 0.00875*** | 0.0194*** | 0.00823*** |
|  | (0.0102) | (0.00247) | (0.00547) | (0.00233) |
| Switzerland | -0.0357*** | 0.00860*** | 0.0190*** | 0.00809*** |
|  | (0.0101) | (0.00243) | (0.00537) | (0.00228) |
| Belgium | -0.0257*** | 0.00619*** | 0.0137*** | 0.00582*** |
|  | (0.00990) | (0.00238) | (0.00527) | (0.00226) |
| Czech Republic | 0.171*** | -0.0412*** | -0.0911*** | -0.0387*** |
|  | (0.0124) | (0.00304) | (0.00675) | (0.00306) |
| Wave 4 (year 2010) | -0.0286*** | 0.00687*** | 0.0153*** | 0.00651*** |
|  | (0.00482) | (0.00117) | (0.00257) | (0.00111) |
| Wave 5 (year 2013) | -0.0130** | 0.00312** | 0.00694** | 0.00296** |
|  | (0.00652) | (0.00158) | (0.00347) | (0.00148) |
| N (Observations) | 45,384 | 45,384 | 45,384 | 45,384 |
|  |  |  |  |  |

Clustered standard errors at household level in parentheses. *** p<0.01, ** p<0.05

We control for age, gender, education, marital status and household income.
